# Supplementary material for: Vaccine effectiveness and duration of protection against symptomatic infections and severe Covid-19 outcomes in adults aged 50 years and over, France, January to mid-December 2021
Source: Glob Epidemiol. 2022 May 27;4:100076. doi: 10.1016/j.gloepi.2022.100076 (PMC9135646; doi:10.1016/j.gloepi.2022.100076)
Supplement: Supplementary file 1 — Supplementary material [file mmc1.docx]

**Appendix. Supplementary materials**

**Appendix 1** • **Milestones of the French sanitary crisis and its management**

| **Context** |  |  | |
| --- | --- | --- | --- |
| 24/01/2020 |  | First confirmed Covid-19 case in France | |
| 14/02/2020 |  | First Covid-19 death in France | |
| **Lockdowns** |  |  | |
| 17/03/2020 | 10/05/2020 | First lockdown | |
| 11/05/2020 | 01/06/2020 | First phase of lifting of lockdown restrictions (gradual reopening according to local health situations) | |
| 02/06/2020 | 21/06/2020 | Second phase of lifting of lockdown restrictions | |
| 17/10/2020 | 29/10/2020 | Curfew in Île-de-France and in eight metropolitan areas between 9 p.m. and 6 a.m. | |
| 30/10/2020 | 14/12/2020 | Second lockdown | |
| 15/12/2020 | 15/01/2021 | Curfew between 8 p.m. and 6 a.m. throughout metropolitan France | |
| 16/01/2021 | 02/04/2021 | Curfew between 6 p.m. and 6 a.m. throughout metropolitan France | |
| 03/04/2021 | 02/05/2021 | Third lockdown | |
| 03/05/2021 | 29/06/2021 | Gradual lifting of the lockdown restrictions, end of travel restrictions | |
| *03/05/2021* | *18/05/2021* | *Curfew between 7 p.m. and 6 a.m.* | |
| *19/05/2021* | *08/06/2021* | *Curfew between 9 p.m. and 6 a.m.* | |
| *09/06/2021* | *29/06/2021* | *Curfew between 11 p.m. and 6 a.m.* | |
| **Schools closures** | |  |  |
| 16/03/2020 | 10/05/2020 | Closure of all nurseries and schools | |
| 11/05/2020 | 02/06/2020 | Gradual reopening of schools | |
| 05/04/2021 | 25/04/2021 | Closure of schools | |
| 26/04/2021 | 02/05/2021 | Closure of colleges and high schools | |
| **Activity restrictions** | |  | |
| 29/02/2020 |  | Prohibition of public gatherings of more than 5,000 people; in the most affected municipalities,  all collective gatherings are prohibited, schools are closed and it is recommended to limit travel | |
| 14/03/2020 |  | Closure of so-called “non-essential” businesses and  establishments open to the public (restaurants, bars, cinemas, nightclubs, etc.) | |
| 17/03/2020 |  | Ban on leaving the house, except for certain reasons requiring a daily covid travel certificate | |
| 17/03/2020 |  | Closure of the external European Union borders | |
| 11/05/2020 |  | Wearing a face mask is mandatory in public transport | |
| 01/07/2020 |  | Gradual reopening of borders | |
| 20/07/2020 |  | Wearing a face mask is mandatory in closed public places | |
| 27/08/2020 |  | Wearing a face mask is mandatory in all closed places | |
| 30/10/2020 |  | Ban on leaving the house, except for certain reasons requiring a daily covid travel certificate | |
| 30/10/2020 |  | Closure of so-called “non-essential” businesses and establishments open to the public  (restaurants, cafes, cinemas, nightclubs, etc.) | |
| 28/11/2020 |  | Reopening of shops (except bars, restaurants and sports halls) | |
| 31/01/2021 |  | Closure of the external European Union borders | |
| 03/04/2021 |  | Limitation of travel to a radius of 10 kilometres | |
| 03/04/2021 |  | Closure of so-called “non-essential” businesses | |
| 19/05/2021 |  | Reopening of terraces (in cafes, bars and restaurants), shops and cultural and sports venues  (with restrictions) | |
| 09/06/2021 |  | Reopening of cafes, bars and restaurants, easing of restrictions | |
| 09/06/2021 |  | Reopening of borders, subject to conditions depending on the country | |
| **Tests, vaccination, health pass** | | | |
| 27/01/2020 |  | Development of a screening test (Pasteur) | |
| 06/04/2020 |  | Beginning of screening of vulnerable people | |
| 12/05/2020 |  | SI-DEP centralizes test results in a national database | |
| 28/05/2020 |  | Test reimbursed by the French health insurance on prescription | |
| 25/07/2020 |  | Test reimbursed by the French health insurance without medical prescription | |
| 17/10/2020 |  | Deployment of rapid antigenic tests | |
| 27/12/2020 |  | Beginning of the vaccination campaign (residents and staff of  accommodation establishments for the elderly) | |
| 02/01/2021 |  | Opening of vaccination to health workers, caregivers and firefighters caregivers aged 50 or over | |
| 18/01/2021 |  | Opening of vaccination to persons aged 75 or over, then gradually to younger ages | |
| 06/02/2021 |  | Opening of vaccination to health workers, caregivers and firefighters | |
| 19/02/2021 |  | Opening of vaccination to persons aged 50 to 64 with comorbidities | |
| 02/03/2021 |  | Opening of vaccination to persons aged 65 to 74 with comorbidities | |
| 27/03/2021 |  | Opening of vaccination to persons aged 70 or over | |
| 12/04/2021 |  | Opening of vaccination to persons aged 55 or over | |
| 01/05/2021 |  | Opening of vaccination to all adults with comorbidities | |
| 10/05/2021 |  | Opening of vaccination to persons aged 50 or over | |
| 31/05/2021 |  | Opening of vaccination to all adults without condition | |
| 09/06/2021 |  | Health pass is mandatory for public gatherings of more than 1,000 people | |
| 15/06/2021 |  | Opening of vaccination to 12-17 years old | |
| 21/07/2021 |  | Extension of the use of the health pass to public gatherings off more than 50 people | |
| 09/08/2021 |  | Extension of the use of the health pass to all places of sociability without size criteria, to  interregional public transport, etc. | |
| 01/09/2021 |  | Opening of booster dose to persons aged 65 or over, elderly home residents, persons with comorbidities  six months after the initial complete vaccination | |
| 15/09/2021 |  | Mandatory vaccination for health workers and certain professions dealing with people | |
| 15/10/2021 |  | End of reimbursement of tests by the French health Insurance,  except on prescription and for vaccinated persons and minors | |
| 27/11/2021 |  | Opening of booster dose to all eligible adults and eligibility for the booster vaccination is lowered  to five months after the initial complete vaccination | |
| 28/12/2021 |  | Eligibility for the booster vaccination is lowered to three months after the initial complete vaccination | |

**Appendix 2** • **Description of the study population**

Table 1. • Descriptive characteristics of the sample, data collected from January 1^st^ to December 12, 2021.

|  | **Symptomatic negatives** | **Symptomatic positives** | **Hospitalized** |
| --- | --- | --- | --- |
| Number of persons | **N=1,475,037** | **N=437,694** | **N=45,802** |
| **Characteristic** |  |  |  |
| **Sex — no. (%)** |  |  |  |
| Female | 848,818 (57.5) | 226,306 (51.7) | 18,592 (40.6) |
| Male | 626,208 (42.5) | 211,387 (48.3) | 27,210 (59.4) |
| Missing data | 11 (<0.1) | 1 (<0.1) | 0 |
| **Age — no. (%)** |  |  |  |
| 50-64 yr | 815,682 (55.3) | 276,597 (63.2) | 14,323 (31.3) |
| 65-74 yr | 348,718 (23.6) | 95,273 (21.8) | 11,733 (25.6) |
| 75-84 yr | 174,709 (11.8) | 39,127 (8.9) | 9,690 (21.2) |
| ≥ 85 yr | 135,928 (9.2) | 26,697 (6.1) | 10,056 (22.0) |
| **Comorbidity — no. (%)** |  |  |  |
| No | 289,793 (19.6) | 162,870 (37.2) | 12,051 (26.3) |
| Yes | 1,185,244 (80.4) | 274,824 (62.8) | 33,751 (73.7) |
| **One dose — no. (%)** |  |  |  |
| No | 812,653 (55.1) | 291,036 (66.5) | 36,345 (79.4) |
| Yes | 662,384 (44.9) | 146,658 (33.5) | 9457 (20.6) |
| **One dose type — no. (%)** |  |  |  |
| Comirnaty (Pfizer/BioNTech) | 471,622 (71.2) | 100,159 (68.3) | 6,597 (69.8) |
| Spikevax (Moderna) | 55,554 (8.4) | 8,314 (5.7) | 605 (6.4) |
| Vaxzevria (AstraZeneca) | 121,270 (18.3) | 32,864 (22.4) | 1,865 (19.7) |
| Janssen | 13,938 (2.1) | 5,321 (3.6) | 390 (4.1) |
| **Two doses — no. (%)** |  |  |  |
| No | 970,735 (65.8) | 330,638 (75.5) | 41,262 (90.1) |
| Yes | 504,302 (34.2) | 107,056 (24.5) | 4,540 (9.9) |
| **Two doses type— no. (%)** |  |  |  |
| Comirnaty (Pfizer/BioNTech) | 390,933 (77.5) | 78,262 (73.1) | 3,373 (74.3) |
| Spikevax (Moderna) | 47,118 (9.3) | 6,116 (5.7) | 278 (6.1) |
| Vaxzevria (AstraZeneca) | 66,251 (13.1) | 22,678 (21.2) | 889 (19.6) |

Figure 6 • Daily counts of controls, symptomatic and hospitalized cases (averaged over the last 7 days), data collected from January 1^st^ to December 12, 2021

**
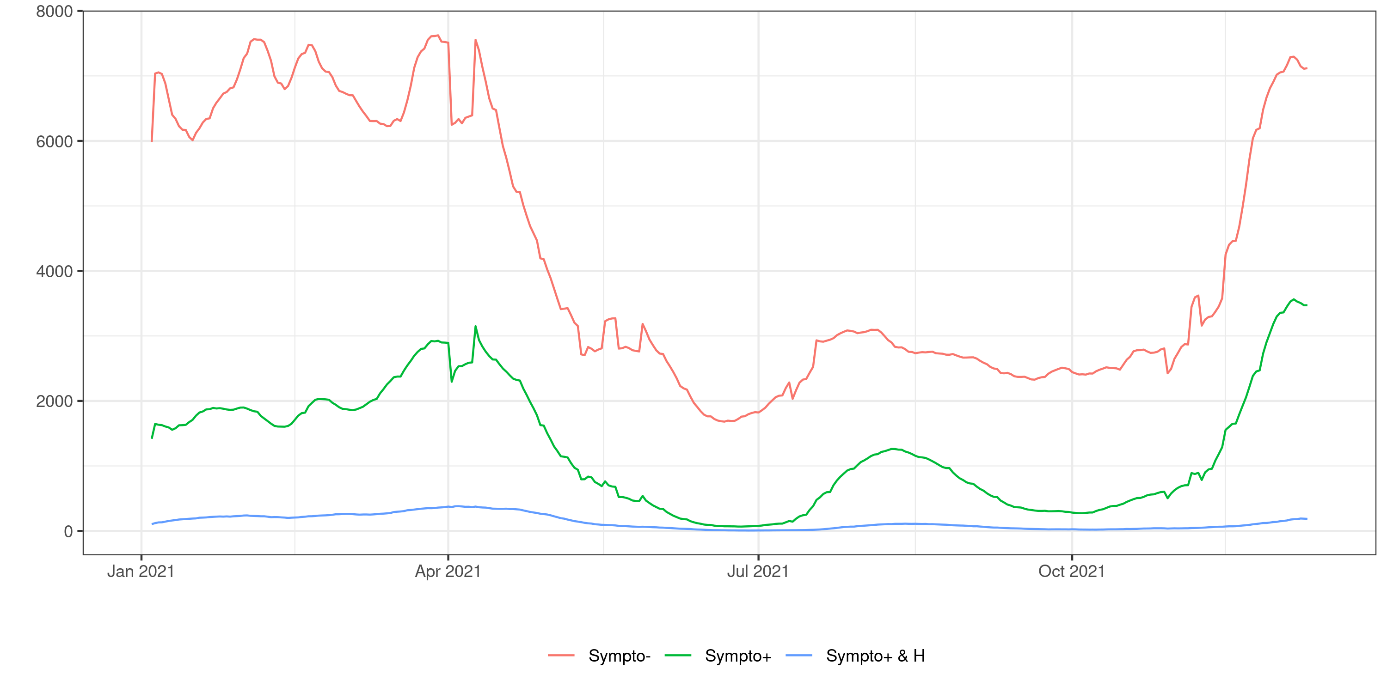
**

Abbreviations: Sympto+ (cases): symptomatic individuals with a laboratory confirmed SARS-CoV-2 infection (cases); Sympto+ & H (hospitalized cases): symptomatic individuals with a laboratory confirmed SARS-CoV-2 infection and hospital admission for Covid-19. Sympto- (controls): individuals with symptoms non-related to SARS-CoV-2 infection.

**Appendix 3 •**

Table 2. • Covid-19 vaccine effectiveness (in %) against symptomatic infections and hospitalizations among persons aged 50 years or over, according to the time elapsed since the receipt of each vaccine dose, data collected from January 1^st^ to December 12, 2021.

| **Age** | **50 years or over** | | **50 years and older With Comorbity** | |
| --- | --- | --- | --- | --- |
| **Vaccination status** | VE(S+) | VE(H+) | VE(S+) | VE(H+) |
| D1: 0-14 | -20.8 (-23.3--18.3) | 15 (11.1-18.8) | -5.5 (-8.5--2.4) | 28 (24.2-31.7) |
| D1: 15-21 | 26.2 (23.9-28.4) | 59.9 (56.8-62.9) | 28.5 (25.5-31.6) | 61.6 (58.4-64.9) |
| D1: 22-28 | 44.5 (42.5-46.6) | 76 (73.7-78.3) | 44.6 (41.9-47.3) | 76.3 (73.8-78.9) |
| D2: 0-7 | 50.4 (47.4-53.4) | 76.7 (73-80.4) | 57.6 (54.0-61.2) | 80 (76.5-83.5) |
| D2: 8-14 | 70 (68.1-71.8) | 84.5 (82.1-87.0) | 71 (68.6-73.5) | 85.6 (83-88.2) |
| D2: 15-30 | 82.4 (81.5-83.2) | 90.9 (89.6-92.2) | 82.9 (81.7-84.1) | 91 (89.5-92.4) |
| D2: 1M-2M | 76.7 (76-77.4) | 94.1 (93.4-94.8) | 78.4 (77.4-79.3) | 94.5 (93.9-95.2) |
| D2: 2M-3M | 70.5 (69.7-71.4) | 93.5 (92.8-94.2) | 69.9 (68.5-71.2) | 93.2 (92.4-94) |
| D2: 3M-4M | 65.7 (64.8-66.7) | 92.7 (92.0-93.4) | 66 (64.6-67.4) | 92.5 (91.6-93.3) |
| D2: 4M-5M | 56.2 (55.2-57.3) | 91.7 (91.0-92.4) | 56.2 (54.5-57.9) | 91.4 (90.6-92.2) |
| D2: 5M-6M | 53.7 (52.6-54.8) | 92.2 (91.6-92.8) | 49.6 (47.8-51.5) | 91.2 (90.4-91.9) |
| D2: > 6M | 53.1 (51.9-54.3) | 89.8 (89.1-90.6) | 47 (44.9-49.0) | 88.1 (87.0-89.1) |
| DR: 1-7 | 56.6 (54.3-59) | 91.3 (89.7-93.0) | 51.7 (47.9-55.5) | 90.2 (88.1-92.2) |
| DR > 7 | 91.9 (91.6-92.3) | 98.7 (98.5-99.0) | 90.5 (89.9-91.1) | 98.4 (98.2-98.7) |

Table 3. • Covid-19 vaccine effectiveness (in %) by age group against symptomatic infections and hospitalizations among persons aged 50 years or over, according to the time elapsed since the receipt of each vaccine dose, data collected from January 1^st^ to December 12, 2021.

| **Age** | **50-64 yr** | | **65-74 yr** | | **75-85 yr** | | **≥ 85 yr** | |
| --- | --- | --- | --- | --- | --- | --- | --- | --- |
| **Vaccination status** | VE(S+) | VE(H+) | VE(S+) | VE(H+) | VE(S+) | VE(H+) | VE(S+) | VE(H+) |
| D1: 0-14 | -17.2 (-20.4--14) | 13.3 (5.8-20.8) | -23.7 (-29.2--18.2) | 6.5 (-2.1-15.2) | -29.9 (-37.7--22) | -4.9 (-15.5-5.6) | -20.5 (-28.8--12.2) | 2.0 (-8.4-12.4) |
| D1: 15-21 | 33.0 (30.1-35.9) | 72.2 (67.2-77.3) | 25.2 (20.4-30) | 56.7 (50.1-63.2) | 15.9 (9-22.9) | 46.0 (37.8-54.2) | 15.9 (7.5-24.3) | 39.1 (29.3-49) |
| D1: 22-28 | 47.0 (44.3-49.8) | 83.0 (78.8-87.1) | 45.8 (41.6-50) | 80.6 (76.3-84.9) | 42.9 (37.4-48.5) | 68.6 (62.6-74.6) | 23.4 (14.6-32.2) | 52.3 (43.2-61.5) |
| D2: 0-7 | 51.0 (46.9-55.2) | 82.5 (75.4-89.5) | 56.6 (50.7-62.6) | 86.6 (81.1-92.2) | 49.3 (41.5-57.1) | 73.0 (64.8-81.2) | 55.6 (47.1-64.2) | 60.7 (49-72.5) |
| D2: 8-14 | 71.4 (69.0-73.8) | 80.2 (74.1-86.2) | 73.5 (69.8-77.3) | 82.7 (76.9-88.5) | 67.4 (62.2-72.5) | 82.3 (76.6-88) | 68.4 (62.8-74) | 85.0 (80-90.1) |
| D2: 15-30 | 81.1 (79.9-82.2) | 91.8 (89.4-94.2) | 83.8 (81.9-85.6) | 89.1 (85.9-92.3) | 86.4 (84.4-88.5) | 92.6 (90.2-94.9) | 84.7 (82.2-87.2) | 89.8 (86.7-92.8) |
| D2: 1M-2M | 75.9 (75.0-76.8) | 95.4 (94.4-96.5) | 75.6 (74-77.2) | 94.0 (92.6-95.4) | 82.5 (80.8-84.3) | 93.8 (92.3-95.3) | 82.3 (80.2-84.4) | 92.0 (90.2-93.8) |
| D2: 2M-3M | 71.4 (70.4-72.4) | 95.5 (94.5-96.5) | 70.1 (68.3-71.9) | 92.6 (91.2-94.1) | 71.7 (68.7-74.6) | 90.1 (87.8-92.5) | 75.9 (72.7-79.1) | 90.7 (88.4-93.1) |
| D2: 3M-4M | 68.4 (67.4-69.5) | 94.6 (93.6-95.7) | 62.8 (60.7-64.9) | 92.4 (91-93.8) | 63.4 (59.9-66.8) | 88.4 (86.1-90.8) | 60.9 (56-65.8) | 82.7 (78.9-86.6) |
| D2: 4M-5M | 60.3 (59.1-61.6) | 94.7 (93.7-95.7) | 57.1 (54.9-59.3) | 91.0 (89.5-92.5) | 52.3 (48.2-56.3) | 87 (84.6-89.4) | 38.2 (31-45.4) | 72.1 (66.6-77.5) |
| D2: 5M-6M | 59.3 (58.1-60.6) | 95.5 (94.7-96.3) | 52.5 (50.2-54.9) | 92.4 (91.2-93.5) | 44.1 (39.7-48.5) | 82.6 (79.9-85.4) | 30.4 (23-37.8) | 73.2 (68.3-78.1) |
| D2: > 6M | 58.6 (57.2-60) | 94 (92.9-95.2) | 52.8 (50.2-55.4) | 90.0 (88.3-91.6) | 36.9 (32-41.8) | 82.2 (79.5-85) | 27.4 (20-34.8) | 69.8 (64.8-74.8) |
| DR: 1-7 | 64.8 (62-67.6) | 95.7 (93.1-98.3) | 53.4 (49-57.9) | 93.1 (90.4-95.8) | 38.3 (28.9-47.6) | 85.8 (80.7-91) | 37.3 (21.5-53) | 62.8 (47.9-77.8) |
| DR > 7 | 93.0 (92.3-93.6) | 98.8 (98.0-99.6) | 92.5 (91.8-93.1) | 99.0 (98.7-99.4) | 89.9 (88.8-90.9) | 98.2 (97.7-98.7) | 89.0 (87.5-90.5) | 97.3 (96.5-98.1) |

Table 4. • Covid-19 vaccine effectiveness (in %) by variant of concern against symptomatic infections and hospitalizations among persons aged 50 years or over, according to the time elapsed since the receipt of each vaccine dose, data collected from January 1^st^ to December 12, 2021.

| **Variant** | **Original Strain** | | **Alpha** | | **Beta/Gamma** | | **Delta** | |
| --- | --- | --- | --- | --- | --- | --- | --- | --- |
| **Vaccination status** | VE(S+) | VE(H+) | VE(S+) | VE(H+) | VE(S+) | VE(H+) | VE(S+) | VE(H+) |
| D1: 0-14 | -32.6 (-43.5--21.7) | 1.7 (-16.7-20.1) | -23.9 (-27.6--20.2) | 4.8 (-1.3-10.8) | -20.1 (-33.7--6.4) | 2.2 (-20.4-24.7) | 4.5 (-0.2-9.2) | 16.7 (6.0-27.4) |
| D1: 15-21 | 24.0 (14.3-33.6) | 61.3 (48.2-74.4) | 26.5 (23.2-29.8) | 56.6 (51.9-61.3) | 4.6 (-11.2-20.4) | 40.8 (19.3-62.4) | 38.9 (34.6-43.2) | 63.3 (55.4-71.3) |
| D1: 22-28 | 49.4 (41.7-57.1) | 69.6 (57.4-81.8) | 43.8 (40.8-46.8) | 75.7 (72.3-79.2) | 30.6 (17.3-43.8) | 55.8 (36.8-74.7) | 51.3 (47.4-55.2) | 79.9 (73.9-85.8) |
| D2: 0-7 | 64.6 (51.6-77.6) | 80.8 (62.5-99.2) | 58.4 (53.9-62.9) | 79.4 (74.2-84.7) | 28.3 (3.7-52.9) | 50.7 (17.3-84.1) | 48.4 (43.7-53.2) | 79.3 (72.7-85.9) |
| D2: 8-14 | 82.3 (75.5-89.2) | 93.6 (86.8-100.4) | 75.6 (72.7-78.6) | 87.9 (84.3-91.4) | 57.7 (42.0-73.4) | 79.2 (59.1-99.3) | 69.4 (66.9-72.0) | 80.6 (75.8-85.3) |
| D2: 15-30 | 91.9 (88.3-95.5) | 95.8 (91.6-100) | 91.3 (90.1-92.5) | 93.7 (91.8-95.6) | 84.2 (78.2-90.3) | 98.2 (95.7-100.8) | 78.6 (77.4-79.9) | 89.3 (87.2-91.4) |
| D2: 1M-2M | 84.5 (78.5-90.6) | 97.2 (93.1-101.3) | 87.0 (85.8-88.3) | 95.2 (94-96.4) | 68.0 (59.1-76.9) | 90.6 (83.3-97.8) | 74.0 (73.1-74.8) | 94.1 (93.3-95.0) |
| D2: 2M-3M | 88.7 (78.9-98.5) | 93.3 (78.7-107.9) | 84.0 (81.8-86.2) | 96.9 (95.4-98.3) | 61.2 (45.7-76.8) | 96.7 (90.1-103.3) | 68.6 (67.6-69.5) | 92.7 (91.9-93.6) |
| D2: 3M-4M |  |  |  |  |  |  | 64.6 (63.6-65.5) | 92.2 (91.4-93) |
| D2: 4M-5M |  |  |  |  |  |  | 56.3 (55.2-57.4) | 91.5 (90.7-92.2) |
| D2: 5M-6M |  |  |  |  |  |  | 53.8 (52.7-54.9) | 92 (91.4-92.6) |
| D2: > 6M |  |  |  |  |  |  | 52.4 (51.1-53.7) | 89.5 (88.7-90.3) |
| DR: 1-7 |  |  |  |  |  |  | 55.9 (53.5-58.3) | 90.9 (89.1-92.7) |
| DR > 7 |  |  |  |  |  |  | 91.9 (91.5-92.3) | 98.7 (98.5-99.0) |

Delta variant cases are approximated by cases from July 7 to December 12, 2021.

**Appendix 4 •Downward bias due to targeting vulnerable individuals**Presence of comorbidities is derived either from prior knowledge from the health insurance on the person status or because a physician recommended to prioritize a non-eligible (at-the-time) individual during the vaccination campaign. Thus, the latter is rare for unvaccinated persons. An alternative specification excludes from the sample persons who are classified as comorbid on a physician recommendation in order to benefit from the vaccine in priority. In this alternative specification, the odd ratio in the early period post first dose indicates a decreased risk [OR: 0.93 (CI 95% 91-95)] instead of the increase risk observed in the baseline specification. The comparison of our estimates to this alternative may inform on the extent of the downward bias due to targeting individuals on unobserved factors. The downward bias in the vaccine effectiveness against symptomatic diseases is very limited in the first month following the second dose, but could reach six percentage points after four months. In contrast, vaccine effectiveness against hospitalization are very similar in both alternatives.

Figure 9 • Covid-19 vaccine effectiveness against symptomatic infections and hospitalizations among persons aged 50 years or over, according to the time elapsed since the receipt of each vaccine dose, data collected from January 1^st^ to December 12, 2021, when excluding persons prioritized by a physician for vaccine administration.
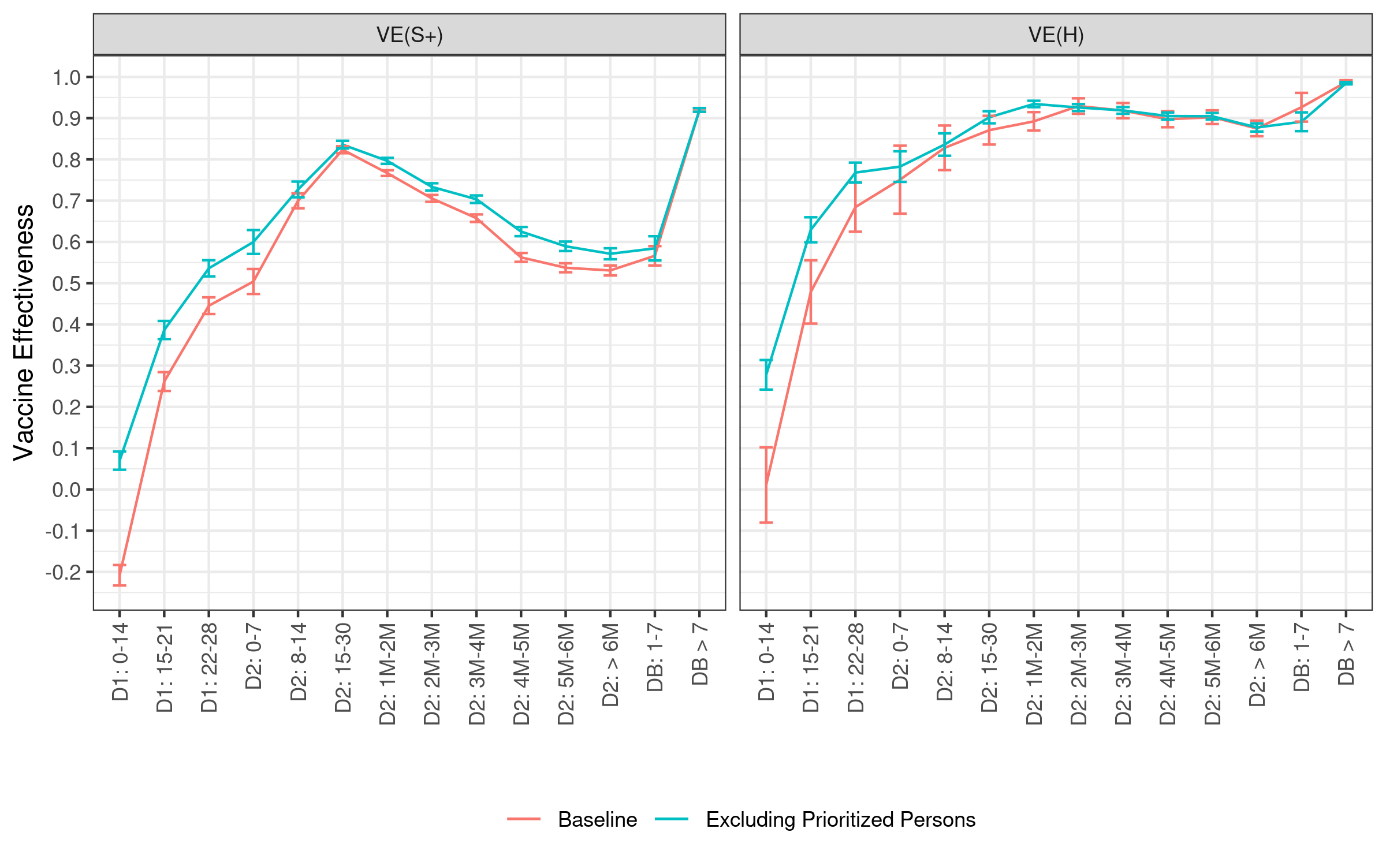


**Appendix 5 • Matching characteristics**

Among persons aged 50 years or over hospitalized for Covid-19 (data source: SI-VIC), 72 % have a linked positive RT-PCR test, collected from fifteen days before admission to the end of their stay (source: SI-DEP) over our analysis period. Among persons aged 50 years or over hospitalized for Covid-19 (data source: SI-VIC), 73 % have a match in the VAC-SI register, which covers nearly all French residents and allows to recover the vaccination status. Among persons aged 50 years reporting symptoms in the last seven days before a RT-PCR test (data source: SI-DEP), 84 % have a match in the VAC-SI register, thus a known vaccination status.
